# Psychometric Properties of a Measure of Borderline Personality Organization in a Spanish Court-Referred Partner-Violent Male Sample

**DOI:** 10.3389/fpsyg.2019.01653

**Published:** 2019-07-12

**Authors:** Natalia Redondo Rodríguez, José Luis Graña Gómez, María Luisa Cuenca Montesino, Marina Julia Muñoz-Rivas

**Affiliations:** ^1^Department of Biological and Health Psychology, Universidad Autónoma de Madrid, Madrid, Spain; ^2^Clinical Psychology Department, Universidad Complutense de Madrid, Madrid, Spain; ^3^Social Psychology Department, Universidad Complutense de Madrid, Madrid, Spain

**Keywords:** validation, psychometric properties, borderline personality organization, borderline personality, intimate partner violence

## Abstract

Borderline personality organization (BPO) is a key personality component of some but not all partner-violent men. The study described in this paper examines the psychometric properties of the borderline personality organization scale (BPO Scale; Oldham et al., 1985) in a Spanish sample of 643 men undergoing court-mandated psychological treatment after conviction for episodes of intimate-partner violence. Three confirmatory factor analyses were carried out first, and the three-factor structure of the BPO scale was then tested. Results for concurrent validity show positive and significant correlations between the subscales and the overall BPO scale, and with other instruments that measure borderline and antisocial personality disorders (ASPDs), and impulsivity. The BPO scale also presents evidence of known-groups validity, since BPO scores decrease with age, and of discriminant validity, as the scale discriminates between participants who do and do not exceed the cutoff point on a borderline personality scale. The BPO Scale is a suitable instrument for evaluating BPO in partner-violent men.

## Introduction

Intimate partner violence (IPV) has become a matter of acute social concern and it is now seen as one of the principal public health issues worldwide, due not only to the scale of the problem but also to the seriousness of its personal, family, social, and legal consequences (DeBoard-Lucas and Grych, 2011; Okuda et al., 2011; Stylianou, 2018). Chief among the adverse outcomes caused by IPV are its effects on victims, including impacts on both physical and mental health, increased risk of depression, anxiety, substance abuse, and suicide (World Health Organization [WHO], 2002). As a phenomenon, IPV takes in many behaviors and is defined by the United Nations (1994) as “any act of gender-based violence that results in, or is likely to result in, physical, sexual or psychological harm or suffering to women, including threats of such acts, coercion or arbitrary deprivation of liberty, whether occurring in public, or private life” (Article 1 of the United Nations Declaration on the elimination of violence against women, 1994). Countless studies have been undertaken in an effort to identify the individual factors distinguishing aggressors, which might help both in predicting aggressive behaviors of this kind, and also provide targets of intervention for treatment programs (Graña et al., 2014, 2017; Jose et al., 2014; Carbajosa et al., 2017; Loinaz et al., 2018).

Among these factors, personality disorders have gained prominence in recent years, as they increase both the risk of an individual’s committing violent acts (Logan and Blackburn, 2009) and of recidivism (Hiscoke et al., 2003). Among all the personality disorders mentioned in the scientific literature on IPV, it is borderline personality disorder (BPD) which is associated with partner-violent men (Holtzworth-Munroe et al., 2000; Jose et al., 2014; Romero-Martínez et al., 2016; Davoren et al., 2017; Mackay et al., 2018). According to the diagnostic criteria established in DSM-5 (American Psychiatric Association, 2014), BPD is theoretically defined by the presence of the following symptoms, among others: (a) alteration of an individual’s personality structure, including disturbance of the sense of identity, intense distortions of self-image, and chronic feelings of emptiness; (b) alteration of affective states, including intense and inappropriate emotional outbursts and anger management issues, as well as general affective instability; (c) behavioral alterations, including suicide attempts and self-harm, as well as extreme impulsivity leading to potentially unsafe behaviors; and (d) alteration of interpersonal relations, including a pattern of unstable yet very intense relationships, constant striving to avoid abandonment, and transitory paranoid ideation.

Meanwhile, the clinical category of borderline personality organization (BPO) is one of the most widely researched risk factors for IPV. BPO was initially described outside the scope of IPV by Gunderson (1984), who listed the following key characteristics: (1) a tendency to maintain intense yet unstable interpersonal relationships; (2) labile sense of self, displaying high levels of anxiety in the face of possible abandonment and very low tolerance to being alone; and (3) intense anger and impulsiveness (Dutton and Starzomski, 1993). In later studies by Dutton (1994, 2007), BPO was identified as a central component of the “abusive personality,” insofar as BPO is a continuum of problems and BPD is merely the most extreme form, while BPO presents a less severe and categorical clinical picture.

One of the most extensively used instruments to assess BPO in partner-violent men is the *Borderline personality organization scale* (Oldham et al., 1985). The scale consists of 30 items distributed across three subscales: Identity Diffusion (10 items), Reality Testing (10 items), and use of Primitive Defenses (10 items). Identity Diffusion is defined as a subjective feeling of inconsistency and problems of self-definition that can in turn lead to emotional disturbance in the context of intimate relationships. Reality Testing refers to an individual’s capacity adequately to perceive reality. Lastly, the Primitive Defenses subscale concerns the defensive mechanisms that an individual unconsciously deploys to minimize the consequences of overly intense emotional situations.

Dutton and Starzomski (1993) evaluated the psychometric characteristics of this instrument for a sample of 120 batterers, correlating the scores of the BPO scale with the borderline pathological personality C scale of the Millon Clinical Multiaxial Inventory-Version 2 (MCMI-II; Millon, 1987), finding positive correlations of 0.71 (Dutton and Starzomski, 1993). They also analyzed the psychological and physical relationship of the BPO with IPV, as measured using the psychological maltreatment of women inventory (PMWI; Tolman, 1989), and the conflict tactics scale (CTS; Straus, 1979). The PMWI assesses different forms of emotional/verbal abuse (e.g., withholding emotional resources, verbal attacks, and behavior that degrades women) and dominance/isolation (e.g., sex roles, demands for subservience, and isolation from resources). Dutton and Starzomski (1993) found significant correlations between the three BPO subscales (identity diffusion, reality testing, and primitive defenses) and the overall BPO scale, and emotional abuse (correlations from 0.50 to 0.55). These authors also used the CTS to assess the frequency and intensity of different dyadic tactics used to resolve conflict, including physical aggression, finding significant correlations between BPO and physical aggression (correlations from 0.21 to 0.33). The reliability of the BPO scale in partner-violent men is reflected in the Cronbach’s alpha values for identity diffusion, primitive defenses, and reality testing, which were 0.85, 0.87, and 0.80, respectively (Tweed and Dutton, 1998).

Numerous researchers have drawn on these early studies to apply the scale to assess BPO in partner-violent men (Dutton et al., 1996; Taft et al., 2004; Stoops et al., 2010), finding that BPO is associated with a greater risk of IPV. However, no study has so far used a confirmatory factor analysis (CFA) to examine the internal structure of the instrument in relation to aggressors of this type. Hence, the main aim of the present study is to examine the characteristics of the BPO scale at the psychometric level among a sample of men convicted of gender violence offenses in Spain. The Spanish comprehensive gender violence protection act (Ley Orgánica 1/2004, 2004) defines gender violence as any act or acts of violence perpetrated by a man against a woman within the scope of an intimate relationship, and as a manifestation of the discrimination, inequality and asymmetrical power relations brought to bear on women by cohabiting, and non-cohabiting intimate partners. Thus, the term “gender violence” will be used for the term IPV in this manuscript as that is the official term used in Spanish law.

The hypotheses guiding this study were: (1) The BPO scale will maintain the same factorial structure as the original study (Oldham et al., 1985) for this sample, consisting of three correlated factors; (2) The three subscales will display adequate reliability; (3) In terms of convergent validity, the BPO scale will correlate with other measures of borderline personality disorder, since BPD is the most severe presentation of BPO (Dutton, 1994, 2007), and with a measure of impulsivity, which is a key aspect of the clinical definition of BPO (Fossati et al., 2004; Liu et al., 2012). It is further expected that the BPO scale will correlate with a measure of antisocial personality disorder (ASPD), since BPD and ASPD are both Cluster B personality disorders and share common characteristics, including disproportionate impulsivity and highly unstable interpersonal relations (Liu et al., 2012). However, it is anticipated that the correlation will be weaker in this case because ASPD and BPD are distinct disorders; (4) In terms of known-groups validity, there will be significant age-based differences in BPO (Jackson and Burgess, 2000; Cohen et al., 2005); and (5) In terms of discriminant validity, it is expected that the scores obtained on the BPO scale will correctly distinguish between individuals who meet the diagnostic criteria for BPD and those who do not.

## Materials and Methods

### Participants

The sample is made up of court-referred partner-violent men from the Madrid Region of Spain convicted of gender violence offenses carrying a prison term of less than 2 years. Though their sentences were suspended, the offenders were ordered by the courts to undergo a psychological treatment program, in accordance with Part IV of Spanish Law 1/2004 on comprehensive gender violence protection measures (Redondo et al., 2017, p.585). The sample included 643 partner-violent men ranging from 18 to 74 years of age (Mean 38.45, *SD* = 10.36). In terms of educational attainment, 41.2% of the sample had completed primary and 42.8% secondary education, while 16% had obtained a university degree. With regard to marital status, 31% of the men in the sample were married or cohabiting, 36.7% were single (i.e., they had no partner at the time of the evaluation), 31.9% were separated or divorced, and 0.6% were widowers. Lastly, 60.5% were Spanish, 29.2% were Central or South American, and 10.2% belonged to other nationalities.

### Instruments

#### Borderline Personality Organization Scale (Oldham et al., 1985)

The BPO scale comprises 30 items and uses a 5-point Likert-type response format with answers ranging from “never true” to “always true.” There are three subscales: identity diffusion (10 items), reality testing (10 items), and use of primitive defenses (10 items). The identity diffusion subscale refers to the fragmentation of mental representations of the self, presenting in the form of grave distortions in the sphere of interpersonal relations, in particular intimate relationships. Examples of the items included in this subscale include: I see myself in totally different ways at different times; I’m afraid of losing myself when I get sexually involved; and I feel empty inside. The reality testing subscale concerns an individual’s ability to maintain a satisfactory perception of reality. BPO individuals often alternate between a clear understanding of reality and distorted or confused perceptions. Examples of the items used in the form to assess this aspect include: I hear things that other people claim are not really there; I’ve had relationships in which I couldn’t feel whether I or the other person was thinking or feeling something; and I believe that things will happen simply by thinking about them. Finally, the primitive defenses subscale deals with the defense mechanisms that the individual unconsciously uses to minimize the repercussions of emotionally charged situations, which might otherwise shake their psychological stability. Items from this subscale include: It is hard for me to trust people because they so often turn against me or betray me. People tend to respond to me by either overwhelming me with love or abandoning me. Uncontrollable events are the cause of my difficulties. In the original studies, the mean score of individuals diagnosed with BPD was 73 on the overall scale compared to 59 for those not diagnosed with BPD. Meanwhile, the reliability scores were 0.92 for identity diffusion, 0.84 for reality testing, and 0.87 for primitive defenses (Oldham et al., 1985).

#### Socio-Demographic Questionnaire

Questions were included to gather participant’s socio-demographic information including age, marital status, nationality, education, and occupation. The information was collected using a semi-structured interview format. Information relating to the offenses committed were gathered from court records.

#### Borderline and Antisocial Personality Disorders

The structured clinical interview for DSM-IV Axis II Personality Disorders (SCID-II; First et al., 1999) was used. This structured interview format is designed to establish the presence or absence of the symptoms listed for different personality disorders in the DSM-IV. In this study, only the items related to the borderline and antisocial personality scales were administered. The test-retest reliability obtained was 0.84 for the antisocial disorder and 0.37 for the borderline disorder.

#### Impulsivity Characteristics

The plutchik impulsivity scale (Plutchik and Van Praag, 1989; Spanish adaptation by Rubio et al., 1998) was used. This scale is made up of 15 items and uses a Likert-type response format based on four alternatives ranging from never to almost always. The scale consists of 4 subscales: Planning capacity (e.g., Do you plan in advance? Are you careful or cautious?). Emotional control (e.g., Do you often become impatient? Do you find it difficult to control your emotions?). Behavior control in relating to eating spending and sex (e.g., Do you spend money impulsively? Do you find it difficult to control your sexual impulses? Do you eat even when you are not hungry?). Other behavior control (e.g., Do you find queuing difficult? Do you often say the first thing that comes into your head?). Reliability was 0.73 in the original study (Plutchik and Van Praag, 1989) and 0.90 in the Spanish adaptation (Rubio et al., 1998).

### Procedure

Prior to beginning this study a Spanish translation and cultural adaptation of the BPO scale was prepared following the guidance of the international tests commission (ITC; Hambleton, 2001). This involved separate translation by two groups of IPV experts with the appropriate cultural background and language skills, before a final consensus version was agreed by both groups. The complete assessment was then carried out before the start of the court-mandated psychological treatment program to which offenders had been assigned. This was the first time that the participants had taken part in a program of this kind. The offense data was collected using court records. From the court, they sent us the sentence of the participants where the facts that had been proven during the trial and for which they had been condemned were recorded. The type of IPV crime was analyzed: psychological, physical or sexual. Each participant attended a 60-min individual session weekly for between 4 and 8 weeks, during which (a) the conditions and goals of the research were explained, and informed consent was obtained in writing (all participants were over the age of 16); (b) socio-demographic data was collected; and (c) the participants completed the questionnaires for the scales described in the Instruments section (counterbalanced self-administration) (Redondo et al., 2017, pp. 585–586).

### Data Analysis

The statistical analyses were carried out using SPSS and AMOS (version 23.0). Cronbach’s alpha was calculated to establish the reliability of the instruments. Pearson’s correlation coefficient was used to evaluate the concurrent validity of the BPO scale. Known-groups validity was assessed by means of a variance analysis to identify age-based differences in the BPO scores and to determine whether or not the borderline personality threshold point had been reached. We also used eta-squared (η*^2^*) to calculate the effect size of the differences found.

We performed a CFA using the AMOS program to analyze the factorial structure of the BPO scale. The goodness-of-fit indices used were CMIN/*df*, goodness of fit index (GFI), adjusted goodness of fit index (AGFI), and root mean-square error of approximation (RMSEA). Values equal to or higher than 0.90 are considered acceptable for GFI and AGFI, whereas values equal to or lower than 0.05 are considered excellent for RMSEA, and values lower than 0.08 are acceptable (Redondo et al., 2017, p. 586).

## Results

### Confirmatory Factor Analysis

Five hypotheses were proposed. The first was that the BPO scale will maintain the same factorial structure as the original study for this sample, consisting of three correlated factors. Three factorial analyses were carried out to test this initial hypothesis: We carried out three CFAs using the maximum likelihood estimation method. Table 1 shows the goodness-of-fit indices of the 3 models. The three-factor model yielded the best goodness-of-fit indices (CMIN/*df* = 2.444; GFI = 0.917; AGFI = 0.901; and RMSEA = 0.047). The GFI and AGFI indices are above 0.90, while the RMSEA index is below 0.05. These data mean that the model that best fits this scale is the three-factor model established in the original study. Therefore, the structure based on three correlated factors is the one that best fits the data obtained from the study. The standardized regression coefficients (standard factor loadings) are shown in Table 2. Scores above 0.40 are considered adequate (Redondo et al., 2017). However, three items were found below this value, although all of them are above 0.37 (i.e., item 9 “I hear things that other people claim are not really there,” item 26 “I am not sure whether a voice I have heard, or something that I have seen, is my imagination or not,” and item 27 “I have heard or seen things when there is no apparent reason for it”). It was therefore decided that these three items should not be removed from the final model given that the other fit indices (see Table 1) were all adequate.

**TABLE 1:** Goodness-of-fit indices for each model.

	**CMIN/*df***	**AGFI**	**RMSEA**	**GFI**
1-factor model	3.479	0.858	0.062	0.878
Model with 3 correlated first-order factors	2.444	0.901	0.047	0.917
Hierarchical 3-factor model	3.508	0.857	0.063	0.878

**TABLE 2:** Three-factor model: Standardized factor loadings for the borderline personality organization (BPO) scale.

		**Squared multiple correlations**	**Factor loading**	**Critical ratio**
	**Item**			
Identity diffusion subscale				
1	I feel like a fake or an imposter, that others see me as quite different at times	0.269	0.518	10.115
5	I see myself in totally different ways at different times	0.426	0.653	11.599
10	I feel empty inside	0.346	0.588	10.936
12	It is hard for me to be sure about what others think of me, even people who have known me well	0.350	0.591	10.975
15	I find it hard to describe myself	0.286	0.535	10.320
17	I don’t feel like myself unless exciting things are going on around me	0.339	0.582	10.864
21	Some of my friends would be surprised if they knew how differently I behave in different situations	0.197	0.443	9.091
24	When I want something from someone else, I can’t ask for it directly	0.187	0.432	8.924
25	I feel I’m a different person at home as compared to how I am at work or school	0.252	0.502	–
Reality testing subscale				
2	I feel almost as if I’m someone else, like a friend or relative or even someone I don’t know	0.202	0.449	10.770
7	I find I do things which get other people upset, and I don’t know why such things upset them	0.437	0.661	15.565
9	I hear things that other people claim are not really there	0.150	0.387	9.314
16	I’ve had relationships in which I couldn’t feel whether I or the other person was thinking or feeling something	0.261	0.511	12.195
19	People see me as being rude or inconsiderate and I don’t know why	0.267	0.517	12.324
20	I can’t tell whether certain physical sensations I’m having are real, or whether I am imagining them	0.235	0.485	11.597
26	I am not sure whether a voice I have heard, or something that I have seen, is my imagination or not	0.139	0.373	8.992
27	I have heard or seen things when there is no apparent reason for it	0.138	0.372	8.950
30	Somehow, I never know quite how to conduct myself with people	0.467	0.683	–
Primitive defenses subscale				
3	It is hard for me to trust people because they so often turn against me or betray me	0.326	0.571	–
4	People tend to respond to me by either overwhelming me with love or abandoning me	0.317	0.563	13.145
6	I act in ways that strike others as unpredictable and erratic	0.358	0.599	12.354
8	Uncontrollable events are the cause of my difficulties	0.297	0.545	11.537
11	I tend to feel things in a somewhat extreme way, experiencing either great joy or intense despair	0.360	0.600	12.371
14	I feel that certain episodes of my life do not count and are better erased from my mind	0.169	0.411	9.189
18	I feel people don’t give me the respect I deserve unless I put pressure on them	0.362	0.601	12.387
22	I find myself doing things which feel okay while I am doing them but which I later find hard to believe I did	0.337	0.580	12.079
28	I feel I don’t get what I want	0.302	0.550	11.605

The second hypothesis was that the three subscales will display adequate reliability. The reliability (Cronbach’s alpha) of the total scale and the three subscales was satisfactory (0.78 for identity diffusion, 0.77 for reality testing, 0.80 for primitive defenses, and 0.91 for the total scale).

### Concurrent Validity

In terms of convergent validity, the third hypothesis of this study is that the BPO scale will correlate with other measures of borderline and antisocial personality, and with a measure of impulsivity. We calculated the correlations between identity diffusion, primitive defenses, and reality testing and different measurements for borderline personality, antisocial personality, and impulsivity. As may be observed in Table 3, all the correlations were either small-to-moderate or moderate, and all were statistically significant (*p <* 0.001).

**TABLE 3:** Means, standard deviations, alpha reliability coefficients, and Pearson’s correlations between BPO subscales and measures of antisocial and borderline personality, and impulsiveness.

**Measure**	**Identity diffusion**	**Reality testing**	**Primitive defenses**	**Total**	***M***	***SD***	**α**
BPO							
Identity diffusion	–				5.582	5.384	0.777
Reality testing	0.763^∗∗∗^	–			3.899	4.342	0.772
Primitive defenses	0.783^∗∗∗^	0.756^∗∗∗^	–		8.159	6.181	0.798
Total	0.924^∗∗∗^	0.896^∗∗∗^	0.934^∗∗∗^	–	17.639	14.643	0.913
SCID II measures of personality							
Borderline	0.656^∗∗∗^	0.603^∗∗∗^	0.641^∗∗∗^	0.691^∗∗∗^	4.063	3.152	0.805
Antisocial	0.292^∗∗∗^	0.297^∗∗∗^	0.299^∗∗∗^	0.312^∗∗∗^	1.700	2.379	0.798
Measures of impulsivity							
Plutchnik	0.549^∗∗∗^	0.521^∗∗∗^	0.535^∗∗∗^	0.582^∗∗∗^	11.412	5.859	0.753

*SCID II, self-report assessment of the diagnostic and statistical manual of mental disorders-IV R personality disorders; Plutchik, plutchik impulsive control scale. ^∗∗∗^*p* < 0.001.*

### Known-Groups Validity

The forth hypothesis was that there will be significant age-based differences in BPO scale. We established three age groups following the criteria described in research on the development of personality over an individual’s lifetime. These studies recommend using certain key decades as transition points to define age groups (Roberts and DelVecchio, 2000; Ullrich and Coid, 2009): men up to 29 years of age (early adulthood), from 30 to 50, and above 50. Significant differences were observed in (a) the overall BPO scale depending on the age group, *F*(2,640) = 5.402, *p* < 0.01, and specifically between the early adult group and the middle-aged group, and the early adult group and the oldest group; (b) identity diffusion, *F*(2,640) = 3.493, *p* < 0.05, and (c) primitive defenses, *F*(2,640) = 3.783, *p* < 0.05. Significant differences were found in both subscales between Groups 1 (≤29 years) and 2 (30–50 years). In all cases, Group 1 (≤29 years) presented higher levels of BPO (see Table 4).

**TABLE 4:** Means, standard deviations, and differences by age in the subscales of the borderline personality organization (BPO) scale.

	**Group 1 (≤29 years) (*n* = 137, 21.3%) *M* (*SD*)**	**Group 2 (30–50 years) (*n* = 422, 65.6%) *M* (*SD*)**	**Group 3 (>50 years) (*n* = 84, 13.1%) *M* (*SD*)**	**Total (*n* = 643) *M* (*SD*)**	***F*(2.640)/ *η^2^***	**Bonferroni**
BPO total	20.679 (16.427)	16.808 (13.914)	16.857 (14.668)	17.639 (14.668)	5.402^∗∗^ 0.02	1>2^∗∗^ 1>3^*^
Identity diffusion	6.898 (6.264)	5.279 (5.039)	4.952 (5.243)	5.582 (5.385)	3.493^*^ 0.01	1>2^*^
Reality testing	4.752 (4.759)	3.628 (4.156)	3.869 (4.420)	3.899 (4.342)	1.748 0.01	
Primitive defenses	9.029 (6.660)	7.900 (6.039)	8.036 (6.017)	8.159 (6.181)	3.783^*^ 0.01	1>2^*^

**η*^2^*, eta-square; ^*^p < 0.05. ^∗∗^*p* < 0.01.

### Discriminant Validity

The last hypothesis of this study was that the scores obtained on the BPO scale will correctly distinguish between individuals who meet the diagnostic criteria for BPD and those who do not. To assess the BPO scale’s ability to distinguish participants who meet the majority of the diagnostic criteria for BPD, as determined from the scores obtained in the SCID II screening questionnaire, we divided the participants into two groups, one including those who passed the instrument’s cut-off point (score equal to or higher than 5) and those who were below the cut-off point. As may be observed in Table 5, the group of participants who passed the cut-off point for BPD diagnosis presented statistically higher scores on the overall BPO scale, *t*(641) = −17.483, *p* < 0.001. Likewise, statistically higher scores were observed in the BPD group in identity diffusion, *t*(641) = −16.982, *p* < 0.001, reality testing, *t*(641) = −14.572, *p* < 0.001, and primitive defenses, *t*(641) = −15.032, *p* < 0.001 (see Table 5).

**TABLE 5:** Means, standard deviations, and differences in the subscales of the borderline personality organization (BPO) scale according to the cut-off point for BPD as measured with the SCID II questionnaire.

	**BPD SCID II below the cut-off point (*n* = 455; 70.8%) *M* (*SD*)**	**BPD SCID II exceeding the cut-off point (*n* = 188; 29.2%) *M* (*SD*)**	**Total (*n* = 643) *M* (*SD*)**	***t*(641)**	***η*^2^**
BPO total	12.295 (10.316)	30.575 (15.497)	17.639 (14.644)	−17.483^∗∗∗^	0.323
Identity diffusion	3.655 (3.789)	10.245 (5.815)	5.582 (5.385)	−16.982^∗∗∗^	0.310
Reality testing	2.508 (2.508)	7.266 (5.272)	3.899 (4.342)	−14.572^∗∗∗^	0.249
Primitive defenses	6.132 (6.132)	13.064 (6.140)	8.159 (6.181)	−15.032^∗∗∗^	0.261

*SCID II, self-report assessment of the DSM-IV R personality disorders. ^∗∗∗^*p* < 0.001.*

## Discussion

Even though the BPO scale is a widely used as an instrument in research involving partner-violent men, no prior research has analyzed the structure of the scale in this population. Our first hypothesis was that the optimum data fit would be achieved in the model proposed in the original study (Oldham et al., 1985). However, we also considered that it would be a necessary first step in adapting the scale for use with a sample of batterers in Spain to establish the goodness-of-fit between the data obtained from this study with all possible factorial models in order to verify that the original study does in fact have the best fit based on empirical data. In the case of the hierarchical model, the hypothesis was that there is an overall BPO score which contains the three subscales proposed in the original study. Therefore, in the present study, we ran the CFA in the three possible models, finding the best fit in the three-factor model, as proposed in the original study. The reliability of the three BPO subscales and the overall scale was above 0.77, and therefore satisfactory in all cases, verifying the second hypothesis regarding the reliability of the scale. Reliability was similar to the scores observed in other studies of batterers (Tweed and Dutton, 1998).

The third hypothesis tested in the study, regarding concurrent validity, was also verified. The BPO scale correlated significantly and positively with BPD, which is the most extreme form of BPO (Dutton, 1994, 2007). We also found significant correlations with impulsivity, a key construct in the clinical characteristics of BPO (Fossati et al., 2004; Liu et al., 2012). Positive and significant correlations were also found between BPO and ASPD, although the scores observed were somewhat lower because these are independent disorders. The association between BPO and ASPD is explained by the fact that both personality disorders belong to Cluster B despite their differing clinical presentations, placing them among the disorders most frequently associated with IPV, with which they share clinical features like emotional instability, impulsivity, and unstable personal relations (Liu et al., 2012; González et al., 2016). These significant and positive correlations between the BPO subscales and other external variables indicate that the BPO scale has good concurrent validity, as had already been shown in previous studies (Dutton and Starzomski, 1993; Dutton, 1994).

Given the importance of age as a variable that explains many personality traits (Zanarini et al., 2005, 2006), we analyzed the relationship between the age of the study participants and the BPO scale. We found no research focusing specifically on age-related changes in the levels of BPO, but we did find studies that analyzed the lifetime course of BPD. Historically, the supposed stability of personality traits and disorders has been considered a key feature. However, more recent studies increasingly suggest that personality disorders vary over a person’s lifetime, undergoing periods of stability and change (Clark, 2005). In this light, it would seem that the general tendency is for the prevalence of personality disorders to decrease with age according to both longitudinal studies (Cohen et al., 2005) and cross-sectional studies (Jackson and Burgess, 2000). Furthermore, the relationship between younger age and personality disorders seems to be especially relevant in the case of Cluster B disorders (Samuels et al., 2002; Coid et al., 2006), in particular BPD (Engels et al., 2003). Our results regarding the relationship between BPO and age provide evidence for the existence of known-groups validity, and they are likewise consistent with earlier findings indicating that BPD traits are more acute before the age of 30 and stabilize increasingly between the ages of 50 and 70 (Roberts and DelVecchio, 2000; Terracciano et al., 2006). The fourth hypothesis proposed, regarding age, was therefore verified.

Finally, the fifth hypothesis, regarding discriminant validity, was also verified. We found that participants who met the diagnostic criteria for BPD scored significantly higher on the three BPO subscales and on the total scale compared to the group of participants who did not meet the diagnostic criteria for BPD. BPO is a continuum of disturbances in which BPD is the pole of greatest severity (Dutton, 1994, 2007), so these results are plausible from a theoretical viewpoint.

This study constitutes an important advance in research into partner-assaultive men. In recent years, numerous papers have shown that aggressors of this kind do not form a single homogeneous group. Rather, various different subtypes exist depending on the severity, frequency and generality of the IPV exhibited, levels of anger and the presence of associated psychopathology (Holtzworth-Munroe and Stuart, 1994; Holtzworth-Munroe et al., 2000; Cavanaugh and Gelles, 2005; Graña et al., 2014). The availability of valid, reliable instruments to measure these characteristics should help with the early identification of the most severe partner-assaultive men, who present with disturbed personality traits and disorders, thereby allowing the adaptation of intervention programs to address their specific needs (Cavanaugh and Gelles, 2005; Stoops et al., 2010). Where aggressors present disturbed personality traits or diagnosed personality disorders, the clinical formulation of each specific case is especially relevant, as is the existence of psychometrically guaranteed assessment instruments to allow evaluation of the core intervention goals. The present study thus underscores the importance of instruments to assess BPO for a range of purposes, including (a) research to establish the risk factors involved in IPV and the profiles and subtypes of aggressors with differential personality traits in order to design and hone different intervention strategies; (b) evaluation of the effectiveness of the psychological treatment programs undertaken with aggressors; and (c) forensic assessments and evaluations, given that the population concerned are convicted offenders serving suspended prison sentences.

This study has certain limitations which should be addressed. In the first place, there are three items in the model with factor scores of less than 0.40, although it was decided to retain them given that the scores for the remaining indices were satisfactory. Also, we only analyzed the data of partner-violent men sentenced to prison terms of less than 2 years. The legal situation of the participants in this study and the possible social desirability attendant upon their responses may therefore have affected their responses. Meanwhile, future research might explore the capacity of the BPO scale to predict recidivism, given the association between BPD and reoffending among partner-violent men (Taft et al., 2004; Romero-Martínez et al., 2016). Lastly, it would be interesting to complete the results of these self-reported scales with interviews designed directly to analyze the diagnostic criteria for BPD and the clinical features of BPO.

## Ethics Statement

This study was approved by the Ethics Commission of the Faculty of Psychology of the University Complutense of Madrid, on May 30, 2009.

## Author Contributions

All authors contributed to revision, read, and approved the submitted version of the manuscript.

## Conflict of Interest Statement

The authors declare that the research was conducted in the absence of any commercial or financial relationships that could be construed as a potential conflict of interest.
